# Disturbed Blood Flow Acutely Increases Endothelial Microparticles and Decreases Flow Mediated Dilation in Patients With Heart Failure With Reduced Ejection Fraction

**DOI:** 10.3389/fphys.2021.629674

**Published:** 2021-03-11

**Authors:** Thiago O. C. Silva, Allan R. K. Sales, Gustavo S. M. Araujo, Guilherme W. P. Fonseca, Pedro G. S. Braga, Diego Faria, Helena N. M. Rocha, Natalia G. Rocha, Marta F. Lima, Charles Mady, Carlos E. Negrão, Maria Janieire N. N. Alves

**Affiliations:** ^1^Heart Institute, University of São Paulo Medical School, São Paulo, Brazil; ^2^D’OR Institute for Research and Education, São Paulo, Brazil; ^3^Department of Physiology and Pharmacology, Fluminense Federal University, Niteroi, Brazil; ^4^School of Physical Education and Sport, University of São Paulo, São Paulo, Brazil

**Keywords:** disturbed blood flow, endothelial microparticles, endothelial function, heart failure, vascular

## Abstract

**Introduction:**

Disturbed blood flow, characterized by high retrograde and oscillatory shear rate (SR), is associated with a proatherogenic phenotype. The impact of disturbed blood flow in patients with heart failure with reduced ejection fraction (HFrEF) remains unknown. We tested the hypothesis that acute elevation to retrograde and oscillatory SR provoked by local circulatory occlusion would increase endothelial microparticles (EMPs) and decrease brachial artery flow-mediated dilation (FMD) in patients with HFrEF.

**Methods:**

Eighteen patients with HFrEF aged 55 ± 2 years, with left ventricular ejection fraction (LVEF) 26 ± 1%, and 14 control subjects aged 49 ± 2 years with LVEF 65 ± 1 randomly underwent experimental and control sessions. Brachial artery FMD (Doppler) was evaluated before and after 30 min of disturbed forearm blood flow provoked by pneumatic cuff (Hokanson) inflation to 75 mm Hg. Venous blood samples were collected at rest, after 15 and 30 min of disturbed blood flow to assess circulating EMP levels (CD42b−/CD31+; flow cytometry).

**Results:**

At rest, FMD was lower in patients with HFrEF compared with control subjects (*P* < 0.001), but blood flow patterns and EMPs had no differences (*P* > 0.05). The cuff inflation provoked a greater retrograde SR both groups (*P* < 0.0001). EMPs responses to disturbed blood flow significantly increased in patients with HFrEF (*P* = 0.03). No changes in EMPs were found in control subjects (*P* > 0.05). Disturbed blood flow decreased FMD both groups. No changes occurred in control condition.

**Conclusion:**

Collectively, our findings suggest that disturbed blood flow acutely decreases FMD and increases EMP levels in patients with HFrEF, which may indicate that this set of patients are vulnerable to blood flow disturbances.

## Introduction

Disturbed blood flow, characterized by high retrograde and oscillatory shear rate (SR), plays a proatherogenic role across the vascular endothelium (Chiu and Chien, 2011; Davies et al., 2013). Arterial regions exposed to disturbed flow, such as bifurcations and curvatures, are extremely vulnerable to the development of atherosclerosis, whereas regions exposed to laminar and moderate SR are protected from this vascular alteration (Chiu and Chien, 2011; Davies et al., 2013). These findings are supported by cell culture and isolated vessel studies that demonstrate endothelial injury and pathologic vascular remodeling induced by disturbed blood flow (Carter et al., 2013). Low SR reduces nitric oxide (NO) bioavailability by decreasing eNOS expression, thereby exposing the endothelium to the atherogenic effect of risk factors (Chatzizisis et al., 2007). In humans, it has been consistently shown that acute elevations to retrograde and oscillatory SR decrease the flow-mediated dilation (FMD) of atherosclerosis-prone and resistant conduct arteries (Gambillara et al., 2006; Schreuder et al., 2014; Thijssen et al., 2015).

Heart failure with reduced ejection fraction (HFrEF) is a complex syndrome, characterized by peripheral vasoconstriction, as a result of an elevated sympathetic nervous system (Rihal et al., 2015) and renin-angiotensin system activity (Taylor, 2012), as well as reduced endothelial function (Katz et al., 1992). These changes greatly contribute to the skeletal myopathy and exercise intolerance in patients with chronic heart failure. However, whether disturbed blood flow activates the endothelial cells or exacerbates the endothelial dysfunction in patients with HFrEF is unknown.

Microparticles are cell-derived vesicles up to 100 nm released after activation or apoptosis of the endothelium or blood cells types (Franca et al., 2015). Specifically, endothelial cell-derived microparticles (EMPs) represent a very important subset of all circulating microparticles (Markiewicz et al., 2013). It was previously reported that increased levels of circulating EMP are an important factor in the development of endothelial dysfunction, a pivotal role in the pathophysiology of cardiovascular diseases (Brodsky et al., 2004). Circulating EMPs stimulate recruitment and adhesion of activated platelets and leukocytes by delivering specific proteins (e.g., growth factors, messenger RNAs, microRNAs, and cell surface receptors), which can modulate the response in the target tissue (Burnouf et al., 2014). The cell-to-cell signaling between EC-CM might also contribute to endogenous and pharmacological pathways (Leucker et al., 2011), altering the biological activity of these cells and influencing, for example, the processes of inflammation, cellular senescence, and apoptosis (Dignat-George and Boulanger, 2011; Diehl et al., 2012; Hulsmans and Holvoet, 2013). Previous studies show that 20–30 min of retrograde and oscillatory SR on the brachial artery increases EMPs in the circulation of healthy subjects (Jenkins et al., 2013; Tremblay et al., 2017) and patients with arterial hypertension (Rocha et al., 2018). Because patients with HFrEF have impaired FMD and elevated circulating EMP levels, indicative of pro-atherogenic endothelial phenotype, it is possible to raise the question that acute elevations in retrograde and oscillatory SR promote an increase in EMPs.

In this study, we sought to determine the effects of disturbed blood flow on the release of EMPs and endothelial function in patients with HFrEF. We hypothesized that (1) acute elevation in retrograde and oscillatory SR would increase circulating EMPs and (2) would decrease FMD in patients with HFrEF.

## Materials and Methods

### Ethical Approval

All experimental procedures and measurements conformed to the Declaration of Helsinki and were approved by the Research Committee of the Heart Institute (SDC: 4374/16/040), the Human Subject Protection Committee at the Clinics Hospital of the University of São Paulo Medical School (CAEE 57991116.4.0000.0068). All patients provided written informed consent before enrollment.

### Samples

A total of 32 subjects (18 patients with HFrEF and 14 age-matched controls) were included in the study. All patients had been on stable heart failure medications for >3 months, had LVEF ≤ 40%, New York Heart Association functional class I–II, optimal medical therapy, and peak oxygen uptake ($\dot{\text{V}}$O_2_) ≤ 20 mL kg^–^1 per min. The exclusion criteria were myocardial infarction within 3 months, unstable angina, acute HF, pacemaker, pulmonary disease, chronic renal disease, peripheral neuropathy, history of stroke, untreated hypo/hyperthyroidism, body mass index (BMI) > 30 kg/m^2^, or history of smoking. Screening for the study consisted of a complete medical history questionnaire, followed by physical examination that included biochemical blood analysis, resting blood pressure (BP) measurement (sphygmomanometry), a resting electrocardiogram, anthropometry (i.e., body weight and height), cardiac function test (echocardiogram), and cardiopulmonary exercise testing.

### Experimental Design

**FIGURE 1:**
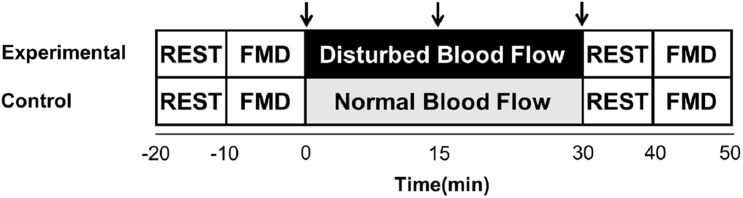
Experimental design for experimental and control sessions. FMD, brachial artery flow-mediated dilation; ↓, venous blood samples.

The study protocol was performed randomly in two sessions, experimental and control (Figure 1). All sessions were conducted in the afternoon, 7 days apart. Before each visit, subjects abstained from alcohol and caffeine for 24 h and from intense physical activity for 48 h. All patients were instructed to continue with their medications during the study, except diuretics for practical reasons, before all measurements.

During the experimental session, the subjects were placed in a supine position, followed by a 10-min quiet resting period. Brachial artery FMD was performed in the left arm. Thereafter, an intravenous catheter was placed into the brachial vein of the same arm and blood samples were collected to evaluate EMPs. Two pneumatic cuffs were placed on the left arm for 30 min, a cuff on the forearm, inflated to 75 mm Hg to produce disturbed blood flow and a proximal cuff positioned 3 cm distal from the axilla, inflated to 40 mm Hg to partially occlude venous flow from the arm, thereby facilitating the trapping of EMPs that would be released during the experiment. Blood samples and blood flow patterns were evaluated at three time points, 0 (before the inflation of the cuffs), and 15 and 30 min (during inflation of the cuffs). Finally, after an interval of 10 min, the FMD was performed again. The control session consisted of the same protocol; however, the cuffs were not inflated to produce disturbed blood flow.

### Measures and Procedures

#### Cardiac Function

Left ventricular ejection fraction (LVEF) was determined by two-dimensional echocardiography by Simpson’s biplane method. All parameters, LVEF, LV end-diastolic diameter (LVEDD), LV end-systolic diameter (LVESD), LV end-systolic volume (LVESV), and LV end-diastolic volume (LVEDV) were evaluated by an echocardiographer (Marwick, 2015).

#### Cardiopulmonary Exercise Test

Maximal exercise capacity was determined by means of a maximal progressive exercise test on a cycle ergometer for the lower limbs (Micromed – Cardio PC 13) with the ramp protocol, increasing from 5 to 25 W per minute, based on the clinical status of the patients (Myers and Bellin, 2000). Oxygen uptake ($\dot{\text{V}}$O_2_) and carbon dioxide production were determined by means of gas exchange on a breath-by-breath basis in a computerized system (Vmax 229; SensorMedics, Buena Vista, CA, United States). $\dot{\text{V}}$O_2_ peak was defined as the maximum attained $\dot{\text{V}}$O2 at the end of the exercise period. Anaerobic threshold (AT) was determined to occur at the breakpoint between the increase in the carbon dioxide output and $\dot{\text{V}}$O_2_ (V-slope) or the point at which the ventilatory equivalent for oxygen and end-tidal oxygen partial pressure curves reached their respective minimum values and began to rise (Binder et al., 2008). Respiratory compensation was determined to occur at the point at which the ventilatory equivalent for carbon dioxide was lowest before a systematic increase and when end-tidal carbon dioxide partial pressure reached a maximum and began to decrease. $\dot{\text{V}}$O_2__*peak*_ and AT were measured by a cardiologist blinded to randomization. The $\dot{\text{V}}$E/$\dot{\text{V}}$CO_2_ slope was measured by linear regression, incorporating all exercise data.

#### Flow Mediated Dilation

Before and after intervention (disturbed blood flow or normal flow), we measured brachial artery FMD, in accordance to the guidelines (Harris et al., 2010; Thijssen et al., 2011, 2019). FMD was measured in the left arm, with the shoulder abducted at approximately 80° and the forearm supinated. In accordance with the most recent FMD guidelines, an appropriate-sized rapid inflation/deflation pneumatic cuff (E-20 Rapid Cuff Inflator, D.E. Hokanson) was placed around the left forearm, immediately distal to the olecranon process. The brachial images (2–12 cm above the antecubital fossa) were obtained through duplex mode and ultrasound equipment (Vivid E9, General Electric, Horten, Norway) equipped with a 13 MHz linear probe. The contrast resolution, depth, and gain were adjusted to optimize the longitudinal images of the lumen/arterial wall interface. Brachial artery diameter and insonation angle-corrected (≤60°) blood velocity spectra were simultaneously recorded via the pulsed-wave mode at linear frequencies of 13 and 6.0 MHz, respectively. The sample volume was located at the center of the brachial artery and then adjusted to cover vessel width. After this, baseline diameter and blood velocity waveforms were continuously recorded over 60 s. Reactive hyperemia was assessed immediately after relief of 5 min of total ischemia, which was induced by external compression of the arm using an inflatable cuff at 220 mm Hg. After this period, the cuff was rapidly deflated and the changes in artery diameters from baseline were expressed as percentages. The probe location was marked on the skin to guarantee that the FMD was repeated in the same location during the study.

#### Disturbed Blood Flow Intervention

After 10 min of quiet resting, a 30-min intervention to induce disturbed blood flow or normal flow (control) was conducted. Then a pneumatic cuff (Hokanson) was placed around the left forearm and inflated to 75 mm Hg, and a second proximal cuff was positioned 3 cm distal from the axilla and inflated to 40 mm Hg to partially occlude venous flow from the arm. For normal flow measures, the cuff was placed on the same arm, but it was not inflated. Mean SR and the pattern of SR (anterograde, retrograde, and oscillatory) were recorded by 60 s at three different time points, 0 (before the inflation of the cuffs), and 15 and 30 min (during inflation of the cuffs).

#### Endothelial Microparticles

Venous blood samples were collected in vials containing acid citrate dextrose before (baseline) and during (15 and 30 min, respectively) experimental and control sessions. The endothelial microparticle populations CD31^+^/CD42b^–^ were determined by flow cytometry (BD FACS Verse; BD Biosciences; Franklin Lakes, NJ, United States), as previously described (Jenkins et al., 2013; Rocha et al., 2018). Briefly, 50 μL of platelet-poor plasma samples were incubated in the dark with 4 μL of CD31-FITC (BD Biosciences; Franklin Lakes, NJ, United States) and 4 μL of CD42b− APC (BD Biosciences; Franklin Lakes, NJ, United States) for 30 min at 4°C. Samples were diluted with 450 μL of sterile PBS before flow cytometry analysis. Microparticles were determined as events smaller than 0.9 μm (0.9 μm NIST Traceable polystyrene particle beads, Polysciences Inc., Warrington, PA, United States). Flow rate was set on low, and all samples were run for 90 s. TruCount beads (BD Biosciences; Franklin Lakes, NJ, United States) were used to calculate the concentration of microparticles by using (microparticles per microliter of plasma) the following formula according to manufacturer’s instructions: [(number of events acquired/absolute number of TruCount beads) × (total number of TruCount beads per test/total sample volume)].

#### Flow Mediated Dilation and Blood Flow Analysis

The Doppler ultrasound video signal was encoded real-time and captured at a frequency of 30 Hz. The video files were compatible with commercial automated edge-detection and wall-tracking software (Medical Imaging Applications, Coralville, IA, United States), which was used for offline analysis. The initial phase of the software analysis consisted of identifying regions of interest on the optimal portion of the brachial artery image and its blood velocity spectra. R-wave gaiting function was applied to continuously assess brachial artery diameter or blood velocity. FMD was calculated as the percentage rise of this peak diameter from the preceding baseline diameter. After deflation of the cuff, the cumulative SR was determined by a 3-min period through the area under the curve (AUC; s^–1^⋅). But the FMD was normalized by SRAUC until peak vasodilation (peak diameter). FMD reliability was evaluated through the comparison of FMD value (pre intervention) between days. The intraclass correlation coefficient was 0.91 (*P* < 0.05), and the coefficient of variation was 5.5%.

Shear rate was calculated as four times the ratio between mean blood velocity (*V*_*mean*_; in cm/s) and artery diameter (in cm) [i.e., SR = 4 × (*V*_*mean*_/diameter)]. For calculations of antegrade and retrograde SR, antegrade and retrograde mean blood velocities were used, respectively. In addition, oscillatory SR index, a variable that estimates the magnitude of oscillation in the vascular bed was calculated as IRetrograde shearI/(IAntegrade shearI+IRetrograde shearI. Oscillatory SR index values range from 0 to 0.5, with 0 corresponding to unidirectional shear throughout the cardiac cycle, and 0.5 representing pure oscillation with time-average shear equal to 0. Oscillatory SR was expressed in arbitrary units (a.u.). The vascular and EMPs response to experimental sessions are presented as baseline and maneuver (the peak between time points 15 and 30 min). The delta changes at peak were from baseline.

### Statistical Analysis

The Shapiro–Wilk test was used to verify data distribution, and the Mauchly test was used to verify sphericity. The normality and sphericity assumptions were not violated. The Student paired *t*-test was used to compare baseline characteristics between the groups. Two-way repeated-measures ANOVA were used to analyze the EMPs, FMD variables, and SR patterns between experimental and control sessions. Fisher least significant difference (LSD) *post hoc* tests were applied when significant *F*-values were found. Data are presented as mean ± standard error of the mean, percentage, or absolute delta. Significance was set at *P* ≤ 0.05. All analyses were performed using STATISITICA 12.0 software.

## Results

### Baseline Measurements

Heart failure with reduced ejection fraction patients and control subjects were well matched for age, sex, other physical characteristics and BP and heart rate (Table 1). HFrEF patients had lower LDL cholesterol, $\dot{\text{V}}$O_2__*peak*_, and LVEF than control subjects had (*P* < 0.05). In addition, HFrEF patients had higher glucose, LVDD, LVSD, and creatinine compared with control subjects (to all variables, *P* < 0.05). The estimated glomerular filtration rate was similar between groups (*P* = 0.14). All patients were clinically stable, based on normal jugular venous pressure, and absence of orthopnea, body weight gain, pulmonary congestion, hepatomegaly, or lower limb edema. The control subjects were not currently taking any medications, and the relevant medications used by the patients with HFrEF are reported in Table 1.

**TABLE 1:** Baseline characteristics of patients with heart failure with reduced ejection fraction and control individuals.

Variables	HFrEF	Control	*P-*values
*N*	18	14	
Age, year	54.72 ± 1.72	49.00 ± 2.53	0.07
Sex, M/F	16/2	12/2	0.53
BMI, Kg/m^2^	25.50 ± 0.94	30.85 ± 5.44	0.28
VO_2peak,_ ml/Kg/min	15.90 ± 0.84	37.78 ± 5.08	<0.0001
Systolic BP, mmHg	118.00 ± 12.00	124.00 ± 10.00	0.30
Diastolic BP, mm Hg	68.00 ± 11.00	64.00 ± 6.00	0.23
Heart Rate, bpm	69.00 ± 11.00	67.00 ± 8.00	0.65
Total cholesterol, mg/dl	168.27 ± 9.50	196.40 ± 14.36	0.10
HDL cholesterol, mg/dl	47.05 ± 2.90	50.73 ± 3.77	0.44
LDL cholesterol, mg/dl	93.27 ± 8.89	129.10 ± 11.78	0.02
Glucose, mg/dl	124.30 ± 11.23	93.72 ± 2.14	0.04
Triglycerides, mg/dl	131.61 ± 13.30	102.40 ± 13.35	0.15
Serium creatinine, mg/dl	1.20 ± 0.05	1.06 ± 0.03	0.04
eGFR (ml/min/1.73 m^2^)	57.81 ± 0.89	59.55 ± 0.22	0.14
NYHA FC (I/II)	7/11	–	
LVEF, %	26.05 ± 1.36	65.00 ± 0.98	<0.0001
LVDD, mm	69.72 ± 1.79	47.21 ± 0.63	<0.0001
LVSD, mm	62.61 ± 2.06	30.57 ± 0.65	<0.0001
HF etiology, *n*			
Idiopathic	2	–	
Ischemic	7	–	
Hypertensive	3	–	
Chagasic	4	–	
Alcoholic	2	–	
Medications, *n*			
β-blocker	18	–	
ACEI/ARB	17	–	
Spironolactone	16	–	
Diuretics	16	–	

*Data presented as mean ± SEM. HFrEF, heart failure with reduced ejection fraction; BMI, body mass index; Systolic BP, systolic blood pressure; Diastolic BP, diastolic blood pressure; HR, heart rate; HDL cholesterol, high-density lipoprotein cholesterol; LDL cholesterol, low-density lipoprotein cholesterol; NYHA, New York Heart Association Functional Class; LVEF%, left ventricular ejection fraction; LVDD, left ventricular diastolic diameter; LVSD, left ventricular sistolic diameter; eGRF, estimated glomerular filtration.*

Blood flow patterns, FMD, and EMPs at rest are shown in Table 2. There were no differences in diameter, anterograde velocity, retrograde velocity, mean velocity, anterograde SR, retrograde SR, mean SR, and oscillatory SR index between patients with HFrEF and control subjects (*P* > 0.05). Brachial artery FMD % was lower in patients with HFrEF compared with control subjects (*P* < 0.05). EMPs were similar between groups (*P* > 0.05).

**TABLE 2:** Blood flow patterns, endothelial function, and endothelial microparticles baseline measures in patients with heart failure with reduced ejection fraction and control subjects.

Variables	HFrEF (*n* = 18)	Control (*n* = 14)	*P*-value
Diameter, mm	4.71 ± 0.20	4.10 ± 0.30	0.28
Anterograde Vel, cm/s	8.08 ± 0.54	7.82 ± 1.23	0.61
Retrograde Vel, cm/s	−4.02 ± 0.66	−2.50 ± 0.53	0.12
Mean Vel, s^–1^	4.06 ± 0.60	5.15 ± 1.34	0.20
Anterograde SR, s^–1^	71.51 ± 5.12	80.26 ± 7.53	0.34
Retrograde SR, s^–1^	−33.40 ± 5.44	−27.90 ± 3.96	0.45
Mean SR, s^–1^	35.92 ± 4.96	55.49 ± 14.13	0.17
Oscillatory SR Index, au	0.29 ± 0.03	0.270.03	0.61
FMD %	3.24 ± 0.27	5.01 ± 0.41	0.001
EMPs, MP/μL plasma	236.09 ± 33.45	201.09 ± 36.79	0.52

*Data presented as mean ± SEM. HFrEF, heart failure with reduced ejection fraction; Anterograde Vel, anterograde velocity; Retrograde Vel, retrograde velocity; Mean Vel, mean velocity; Anterograde SR, anterograde shear rate; Retrograde SR, retrograde shear rate; mean SR, mean shear rate; Oscillatory SR Index, oscillatory shear rate index; FMD, flow-mediated dilation; EMPs, endothelial microparticles.*

### Disturbed Blood Flow

**FIGURE 2:**
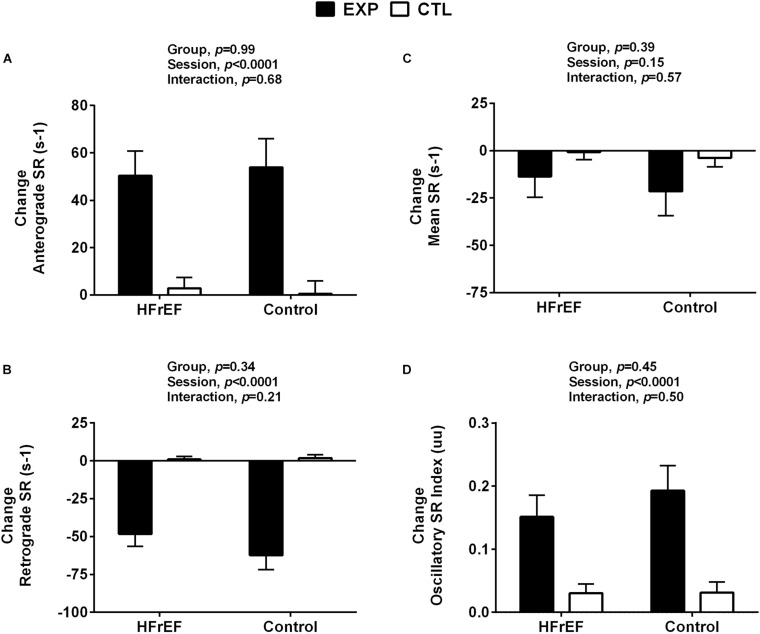
Shear rate responses to control and experimental sessions in patients with heart failure with reduced ejection fraction and healthy subjects. HFrEF, heart failure with reduced ejection fraction; Change anterograde shear rate (Change anterograde SR, Panel **A**); Change retrograde shear rate (Change retrograde SR, Panel **B**); Change mean shear rate (Change mean SR, Panel **C**); Change oscillatory shear rate (Change oscillatory SR changes, Panel **D**). Absolute change was calculated as peak variable – baseline variable.

As expected, the forearm cuff inflation similarly increased retrograde and oscillatory SR index in patients with HFrEF and control subjects (interaction effect, *P* < 0.001; Figures 2B,D, respectively). These variables remained unchanged throughout the control session in both groups (interaction effect, *P* > 0.05). Anterograde SR similarly increased during the experimental session in patients with HFrEF and control subjects (session effect, *P* < 0.001; Figure 2A). Mean SR was not different between sessions and groups (interaction effect, *P* > 0.05; Figure 2C).

### Endothelial Responses to Disturbed Blood Flow

**FIGURE 3:**
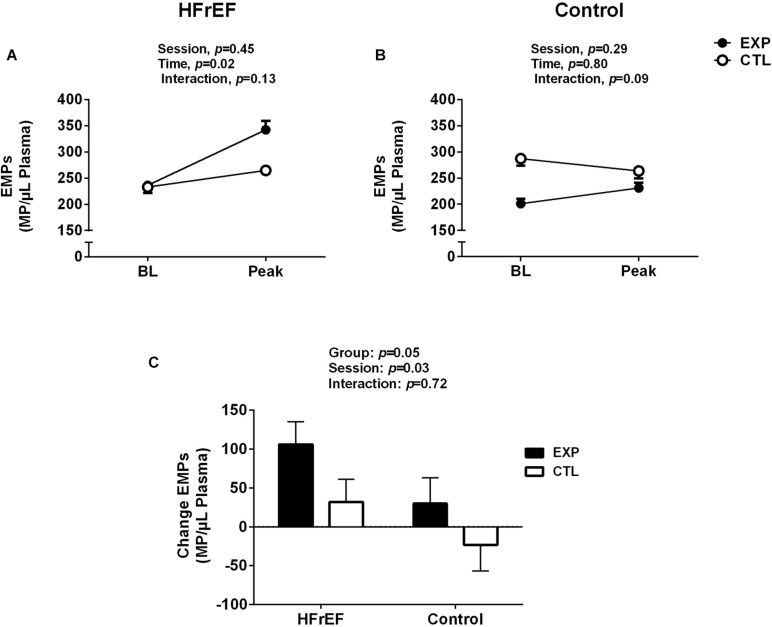
Endothelial microparticles (EMPs) to baseline (BL) and maneuver of disturbed blood flow (experimental session) or normal blood flow (control session) in patients with heart failure with reduced ejection fraction (HFrEF, **A**) and control subjects **(B)**. EMPs responses for disturbed blood flow in patients with HFrEF and control subjects **(C)**. Absolute delta was calculated as peak variable – baseline variable.

To investigate whether disturbed blood flow induces endothelial damage, circulating EMP levels were measured during experimental and control sessions. EMPs significantly increased in patients with HFrEF (time effect, *P* = 0.03, Figure 3A), but not in control subjects in whom EMPs remained unchanged (*P* = 0.80, Figure 3B). The comparisons between groups showed that EMPs response was greater in the patients with HFrEF compared with control subjects (*P* = 0.05, Figure 3C).

### Flow Mediated Dilation

**FIGURE 4:**
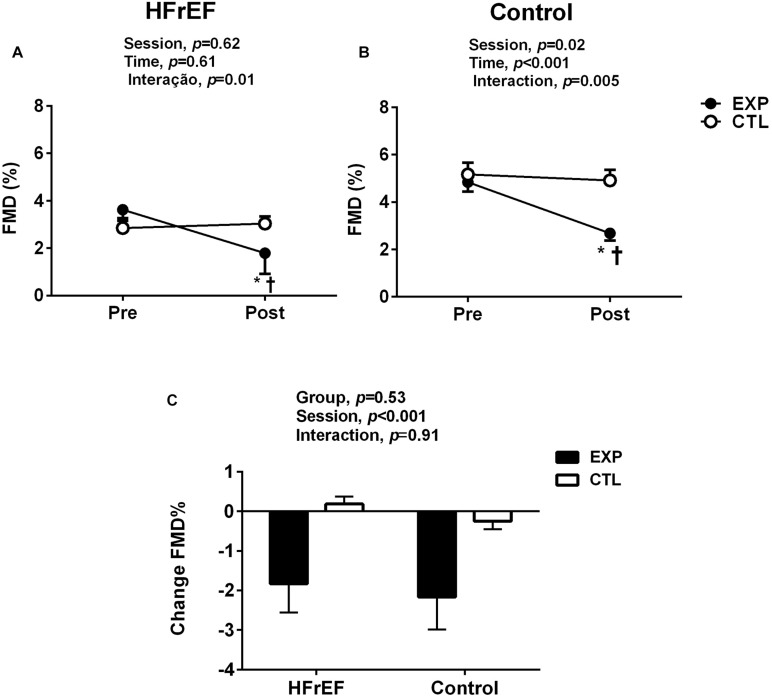
Flow mediated dilation (FMD) before (pre) and (after) post disturbed blood flow (experimental session) or normal blood flow (control session) in patients with heart failure with reduced ejection fraction (HFrEF, **A**) and control subjects and control subjects **(B)**. *P < 0.05 vs. pre and †*P* < 0.05 vs. post in each group. FMD responses for disturbed blood flow in patients with HFrEF and control subjects **(C)**. Absolute delta was calculated as peak variable – baseline variable.

Our analysis revealed that disturbed blood flow similarly reduced the brachial artery FMD % in patients with HFrEF and control subjects (Interaction effect, *P* < 0.001, Figures 4A,B). No changes in FMD % were found in the control session. Delta analysis showed that FMD % response was similar in HFrEF and control (interaction effect, *P* = 0.91, Figure 4C). Resting diameter, AUC SR, and time to peak were not different between experimental and control sessions, and groups (to all variables in both groups, *P* > 0.05, Table 3). Moreover, the disturbed blood flow decreased FMD and FMD %/SRAUC in both groups (*P* < 0.05, Table 3). No changes were observed in this variable in the control session.

**TABLE 3:** Brachial artery flow mediated dilation during experimental and control sessions in patients with heart failure with reduced ejection fraction and control subjects.

Variables	HFrEF (*n* = 18)		Control (*n* = 14)	
	Pre	Post	Pre	Post
**Rest diameter, mm**				
EXP	4.71 ± 0.19	4.68 ± 0.19	4.37 ± 0.14	4.39 ± 0.12
CTL	4.70 ± 0.18	4.68 ± 0.18	4.44 ± 0.13	4.45 ± 0.13
**Peak diameter, mm**				
EXP	4.84 ± 0.20	4.76 ± 1.90	4.58 ± 0.14	4.51 ± 0.12
CTL	4.84 ± 0.19	4.82 ± 0.19	4.68 ± 0.13	4.66 ± 0.13
**FMD, mm**				
EXP	0.16 ± 0.01	0.07 ± 0.04*†	0.20 ± 0.01	0.11 ± 0.01*†
CTL	0.14 ± 0.01	0.14 ± 0.01	0.22 ± 0.02	0.21 ± 0.02
**SRAUC 10^–5^, s^–1^**				
EXP	5.76 ± 0.65	7.64 ± 1.37	6.96 ± 0.99	7.15 ± 1.06
CTL	5.89 ± 0.75	5.93 ± 0.74	6.83 ± 0.78	6.18 ± 1.92
**FMD %/SRAUC10^–5^, s^–1^**				
EXP	8.50 ± 1.41	4.64 ± 1.42 *†	9.27 ± 1.58	4.71 ± 0.90*†
CTL	7.14 ± 1.52	8.39 ± 2.02	9.66 ± 2.10	9.01 ± 1.69
**Time to peak, Sec**				
EXP	68.65 ± 12.55	89.61 ± 9.40	70.64 ± 9.46	80.07 ± 13.12
CTL	79.83 ± 13.25	68.38 ± 9.86	53.14 ± 5.79	65.92 ± 11.55

Data presented as mean ± SEM. HFrEF, heart failure with reduced ejection fraction; EXP, experimental session; CTL, control session; FMD, flow mediated dilation; SRAUC, shear rate area under the curve; FMD %/SRAUC10^–5^, flow mediated dilation normalized by shear rate area under the curve. *P < 0.05 vs. within group difference, and ^†^*P* < 0.05 vs. between group difference.

## Discussion

The purpose of the present study was to examine the acute impact of disturbed blood flow on release of circulating EMPs and brachial artery FMD in HF patients. Our findings indicate that, compared with control subjects, acute elevation in retrograde, and oscillatory SR increase circulating EMP levels in patients with HFrEF.

Disturbed blood flow is associated with endothelial dysfunction (Chiu and Chien, 2011; Davies et al., 2013). Arterial regions exposed to disturbed blood flow, such as bifurcations and curvatures, are extremely vulnerable to the development of atherosclerosis (Chatzizisis et al., 2007; Chiu and Chien, 2011; Davies et al., 2013). Healthy elderly individuals had a great resting retrograde and oscillatory SR in the brachial artery compared with healthy young subjects, which seems to be explained by high alpha-adrenergic vasoconstriction (Casey et al., 2012) and reduced endothelial function (Padilla et al., 2011). We have recently observed that disturbed blood flow in the brachial artery is associated with higher sympathetic outflow to skeletal muscle in young anabolic androgenic steroid users (de Souza et al., 2019). The present study extends the knowledge that acutely augmented retrograde and oscillatory SR is associated with increased EMP production in patients with HFrEF.

EMPs are small vesicles (<1.0 μm) released from the endothelium into the circulation in response to activation or apoptosis of endothelial cells (Markiewicz et al., 2013; Franca et al., 2015). They have been used as a systemic and clinically significant marker of both cardiovascular disease (Franca et al., 2015) and risk of cardiovascular disease (Markiewicz et al., 2013). High levels of circulating EMPs have been described in patients with HF (Rautou et al., 2011; Ivak et al., 2014). To our surprise, the present study does not show differences in resting circulating EMP levels between HFrEF patients and control subjects. One possible explanation is that the patients were taking statins, which may have masked our findings. Statins improve endothelial function and reduce EMPs in patients with cardiovascular disease (Huang et al., 2012; Lins et al., 2014).

In the present study, disturbed blood flow was conducted by forearm cuff inflation at 75 mm Hg for 30 min. This maneuver has been used to manipulate the SR patterns in conduit peripheral arteries (Carter et al., 2013; Schreuder et al., 2014; Thijssen et al., 2015; Rocha et al., 2018). We observed that this hemodynamic maneuver evoked a similar increase in retrograde and oscillatory SR in the brachial artery in patients with HFrEF and control subjects. Although the increase in these responses was similar between groups, EMPs only increased in HFrEF patients. This finding is suggestive of augmented susceptibility to endothelial damage/dysfunction in patients with HFrEF, which may aggravate the clinical condition of these patients. Of note, greater EMP levels have been linked to significant prognostic marker of future cardiovascular complications in the chronic heart failure (Nozaki et al., 2010).

Jenkins et al. (2013) conducted the first study on the effects of disturbed blood flow on the release of EMPs in healthy individuals. The authors reported that 20 min of oscillatory SR elevation clearly increases EMP levels in forearm circulation. Our study does not confirm these findings in control subjects. This controversy is likely explained by differences in the study protocol. Whereas in the Jenkins study the disturbed blood flow was due to a cuff inflated at 200 mm Hg per 20 min, in our study the cuff was set at 75 mm Hg per 30 min, as used previously (Carter et al., 2013; Schreuder et al., 2014; Thijssen et al., 2015; Rocha et al., 2018). In fact, Rocha et al. (2018) also showed that disturbed blood flow using 30 min of forearm cuff inflation at 75 mm Hg did not increase EMPs in control subjects). Also, these different results may suggest that there is a threshold for endothelial activation and release in EMP from healthy endothelium. Thus, future studies should assess the dose-response relationship between increases in oscillatory SR and EMPs release in health and disease.

The exact cellular mechanisms of EMPs release induced by disturbed blood flow are not well established. However, have been shown that several pro-inflammatory and oxidative substances, such as tumor necrosis factor (TNF-α), angiotensin II and reactive oxygen species may contribute for this process (Rautou et al., 2011; Ivak et al., 2014). As heart failure patients have higher circulating levels of angiotensin II and oxidative stress compared to healthy subjects (Katz et al., 1992; Taylor, 2012) it is possible that increase in oscillatory SR, via maneuver (cuff inflated at 200 mm Hg), has elevated these variables, which induced an increase in the releasing of EMPs.

It is well known that patients with HFrEF have endothelial dysfunction (Katz et al., 2005; Shechter et al., 2009; Nozaki et al., 2010). To our knowledge, this is the first study that tested the effects to acute elevations in retrograde and oscillatory SR in patients with HFrEF. Our study shows that HF patients have reduced brachial artery endothelial-dependent function at rest compared to age-matched controls. However, patients with chronic heart failure had decreased brachial artery FMD after disturbed blood flow induction, which is not in accordance with previous findings that evaluated older individuals with endothelial dysfunction (Thijssen et al., 2015). One possible explanation may be the presence of a threshold SR stimulus, which has dose-dependent effects on reducing FMD. Although our study used a cuff inflated to 75 mm Hg, previous studies in older individuals have used a cuff inflated to 60 mm Hg (Schreuder et al., 2015; Thijssen et al., 2015).

Because disturbed blood flow increased EMPs in patients with HFrEF, a greater reduction in the FMD of these patients would be expected. Surprisingly, this was not the case. The reduction of the FMD was similar between patients with HFrEF and age –matched controls. Explanations to this response are outside the scope of our study. However, we speculate that there is a maximum reduction in maneuver-induced endothelial function. Of note, the values for FMD after maneuver were similar between the groups.

### Clinical Implications

Increased EMP levels and reduced endothelial function are associated with an increase in the risk of mortality in patients with chronic heart failure (Shechter et al., 2009; Nozaki et al., 2010). In our study, we demonstrated that acute exposure to disturbed blood flow increases EMPs and reduces endothelial function in patients with HFrEF. This suggests that blood flow disturbances, especially in chronic conditions, may aggravate the cardiovascular complications in this set of patients. Future studies should therefore aim to assess the importance of blood flow patterns in the prognosis of HF patients.

### Limitations

Our study has some limitations. The sample size of our study was relatively modest and should be interpreted with caution. But the results were consistent between and within individuals. The clear dose-response relationship between the independent and dependent variables provides powerful evidence that our findings are robust. Therefore, it is unlikely that a larger number of subjects would have significantly changed the findings of the study. We did not evaluate endothelium-independent function via nitroglycerin, an exogenous donor of NO. Previous studies have shown that stimuli [e.g., mental stress (Spieker et al., 2002) and physical exercise (Vuckovic et al., 2013)] do not change endothelium-independent function. Finally, the sample of our study in both groups was predominantly composed of men. Thus, we cannot extrapolate these results to women. More studies are needed to evaluate the impact of sex on vascular responses to disturbed blood flow.

## Conclusion

Collectively, our findings suggest that disturbed blood flow acutely decreases FMD and increases EMP levels in patients with HFrEF, which may indicate that this set of patients are vulnerable to blood flow disturbances.

## Data Availability Statement

The raw data supporting the conclusions of this article will be made available by the authors, without undue reservation.

## Ethics Statement

The studies involving human participants were reviewed and approved by the Research Committee of the Heart Institute (SDC: 4374/16/040), the Human Subject Protection Committee at the Clinics Hospital of the University of São Paulo 101 Medical School (CAEE 57991116.4.0000.0068). The patients/participants provided their written informed consent to participate in this study.

## Author Contributions

TS and AS conceived experimental designs, performed experiments, interpreted results, and drafted the manuscript. DF, CM, HR, and NR conceived experimental designs, interpreted results, and edited the manuscript. GA, GF, PB, and ML performed experiments and edited the manuscript. CN interpreted results and reviewed/edited the manuscript. MA conceived and designed the study, interpreted results, and reviewed/edited the manuscript. All authors contributed to the article and approved the submitted version.

## Conflict of Interest

The authors declare that the research was conducted in the absence of any commercial or financial relationships that could be construed as a potential conflict of interest.
